# 6.J. Workshop: Precarious employment and its impact on social protections in the EU

**DOI:** 10.1093/eurpub/ckac129.374

**Published:** 2022-10-25

**Authors:** Organised by: EUPHA-SSWH, Chair persons: Ute Bültmann (EUPHA-SSWH), Agnes Meershoek (Netherlands)

## Abstract

The changing nature of work, resulting from technology, globalization, shifts in demographics, and other economic and political forces, poses many potential problems for workers, employers, and society today and for the foreseeable future. Moreover, the COVID-19 pandemic has revealed the importance of social protection systems, e.g. income security for injured or sick-listed workers. The pace of work is increasing, driven by increasing productivity demands and greater use of technology. The nature of employer-employee relationships is also changing rapidly, best exemplified by increases in what has been termed “nonstandard employment arrangements”, such as gig economy work and short-term contracts. However, what is viewed as greater employment flexibility by employers may represent a more precarious job for workers, as compared to pay, benefits and security for those with long-term employment arrangements. Nonstandard employment is typically shorter in duration, leading to more jobs over a lifetime, with the potential for exposure to multiple and simultaneous risks, some of them new, combined with the possibility of more time spent in periods of unemployment or underemployment. Precarious employment has been linked to adverse effects on worker health and well-being. Although these employment arrangements can affect workers of all education and income levels, they disproportionately affect workers in lower socioeconomic strata. One of the major consequences of nonstandard employment arrangements is the potential loss of social protections, including social security, yet this has not been well addressed. Our workshop focuses on the changing work life across different EU member states and beyond, examining different types of precarious employment and their effects on health and social security benefits.

Prof. Ellen MacEachen will focus on how laws, policies and collective agreements in six EU countries, New Zealand and Canada protect the health of low wage and digital platform workers. She will address occupational and public health interventions for these workers in light of health risks related to the COVID-19 pandemic.

Dr. Solveig Osborg Ose will focus on precarious work, sickness absence and risk sharing between employers, employees and social insurance in nine north-western EU countries. Moreover, she will discuss the adaptation of social protection systems to cover all types of employment to avoid increasing inequalities.

Two experts Dr. Anita Tisch and Prof. Angelique de Rijk will reflect on the findings and discuss with the presenters and the audience the challenges, policies and practices needed towards employment and occupational safety and health social protections of precarious workers.

**Key messages:**

• Precarious employment has been linked to adverse effects on worker health and well-being. Workers in lower socioeconomic strata are disproportionately affected.

• A major consequences of nonstandard employment arrangements is the potential loss of social protections, including social security, yet this has to be addressed.

